# Transforming growth factor-β, insulin-like growth factor I/insulin-like growth factor I receptor and vascular endothelial growth factor-A: Prognostic and predictive markers in triple-negative and non-triple-negative breast cancer

**DOI:** 10.3892/mmr.2015.3560

**Published:** 2015-03-27

**Authors:** ABEER BAHHNASSY, MARWA MOHANAD, SABRY SHAARAWY, MANAL F. ISMAIL, AHMED EL-BASTAWISY, ABEER M. ASHMAWY, ABDEL-RAHMAN ZEKRI

**Affiliations:** 1 Molecular Pathology Unit, Pathology Department, National Cancer Institute, Cairo University, Cairo 11796, Egypt; 2 Department of Biochemistry, Faculty of Pharmacy, Misr University for Science and Technology, Cairo 11796, Egypt; 3 Department of Cancer Biology, National Cancer Institute, Cairo University, Cairo 11796, Egypt; 4 Faculty of Pharmacy, National Cancer Institute, Cairo University, Cairo 11796, Egypt; 5 Department of Medical Oncology, National Cancer Institute, Cairo University, Cairo 11796, Egypt

**Keywords:** triple-negative breast cancer, transforming growth factor-β, insulin growth factor/insulin growth factor receptor I, vascular endothelial growth factor, prognosis

## Abstract

In the current study, the prognostic and predictive values of serum transforming growth factor-β1 (TGF-β1), insulin-like growth factor I (IGF-I)/IGF-I receptor (IGF-IR) and vascular endothelial growth factor-A (VEGF-A) were evaluated in triple-negative and non-triple-negative breast cancer (TNBC and non-TNBC). The aim was to identify a group of serological biomarkers and to identify possible candidates for targeted therapy in patients with TNBC and non-TNBC. Protein levels of TGF-β1, IGF-I/IGF-IR and VEGF-A in the serum were measured in 43 TNBC, 53 non-TNBC and 20 normal control participants using quantitative ELISA assays. Results were correlated against standard prognostic factors, response to treatment and survival. TNBC was identified to be associated with poor prognosis and serum levels of VEGF-A and IGF/IGF-IR were significantly higher in the TNBC group compared with the non-TNBC group. IGF-IR and VEGF-A overexpression was observed to be correlated with TGF-β1 expression and all of the markers investigated were associated with metastasis and disease progression. In the multivariate analysis, VEGF-A, IGF-I and IGF-IR were observed to be independent predictors for overall survival, whereas TGF-β1 and lymph node status were identified as independent predictors for disease-free survival. The overall response rate was significantly lower in patients with TNBC and those with high levels of TGF-β1, IGF-I/IGF-IR and VEGF-A. In view of the present results, it was concluded that TGF-β1, IGF-I/IGF-IR and VEGF-A overexpression is associated with the presence of aggressive tumors, which exhibit an increased probability of metastasis, a poor response to treatment and reduced survival rate. This indicates that VEGF-A, IGF-IR and IGF-I have the potential to be used as surrogate biomarkers and are promising candidates for targeted therapy, particularly in patients with TNBC.

## Introduction

Breast cancer (BC) is the most common type of cancer among females worldwide and is the second leading cause of cancer-associated mortality, accounting for 39,520 fatalities among US females during 2011 (1). Recent molecular classification of BC identified subtypes with diverse histopathological features, clinical outcomes and therapeutic implications. Generally, BC is classified into two subgroups, those with tumors that are estrogen receptor-α (ER)-positive; and those that are with ER-negative (2). Triple-negative BC (TNBC) represents a heterogeneous group of tumors that account for 20–25% of all cases of BC. In TNBC, ER, progesterone receptor and human epidermal growth factor 2 (HER2) expression is absent and the cancer is basal-like (expresses basal epithelial cell genes, such as those encoding cytokeratins 5 and 14) (3). Collectively, TNBC represents an aggressive subtype of BC, which is often associated with a high risk of local recurrence, distant metastasis and mortality during the first 3–5 years of follow up (4–6). At present, there is no targeted therapy for TNBC and patients often respond poorly to routine treatment with conventional third generation combination chemotherapy, which is commonly complicated by local recurrence, distant metastasis, frequent recurrence and high mortality rates (4,7). Therefore, there is an increasing demand for novel biomarkers and biological targeted therapies to improve clinical outcome and patient prognosis (8).

A number of circulating tumor proteins have been suggested as prognostic and predictive biomarkers that may be used to assess patients with BC at any stage of the disease, one of which is transforming growth factor-β (TGF-β) (9). Overexpression of TGF-β1 in breast tumors is commonly associated with late-stage disease and/or poor outcome, with frequent occurrence of bone and lung metastases. A study has demonstrated that elevated levels of TGF-β1 and vascular endothelial growth factor (VEGF) promote angiogenesis and stimulate the production of extracellular matrix in the vicinity of the tumor cells. This provides a scaffold for cell proliferation and migration, which facilitates tumor metastasis (10). In addition, treatment with TGF-β1 neutralizing antibodies or receptor kinase inhibitors has been indicated to strongly prevent the development of lung and bone metastases in mouse models of TNBC or basal-like BC. This was attributed to the inhibition of angiogenesis, derepression of antitumor immunity or the reversal of the mesenchymal, motile and invasive phenotypes characteristic of basal-like and HER2-positive BC cells (11).

High serum levels of VEGF-A alone have been commonly associated with unfavorable clinical outcomes, including disease progression, poor response to treatment and reduced survival rates in patients with BC. Therefore, VEGF-A is considered as a prognostic marker and a candidate for targeted therapy in BC, in addition to other solid and hematological malignancies (12,13).

Insulin-like growth factor I (IGF-I) is an important regulator of growth, survival, migration and invasion and is clearly implicated in BC (14). IGF-I stimulation contributes to BC progression via its mitogenic and anti-apoptotic effects on the mammary epithelial cells (15) and additionally protects BC cells from the toxic effects of radio- and chemotherapy (16,17). IGF-IR increases angiogenesis/lymphangiogensis and induces alterations in the integrins and cell adhesion complexes, leading to an increase in cancer cell metastasis (18). Aberrant expression and activity of IGF-I/IGF-IR has been previously detected in proliferative breast tissues in conjunction with significant alterations in cellular morphology, which are associated with cancer progression (15).

Therefore, in the current study the prognostic and predictive values of aberrant serum expression of TGF-β1, VEGF-A and IGF-I/IGF-IR were investigated in patients with TNBC and non-TNBC. To achieve this, the expression levels of the markers in the two groups were correlated with the standard prognostic factors for BC, the response to treatment and the survival rates. It was hypothesized that the results may aid in the further elucidation of the function of these interrelated proteins in the development and progression of TNBC. Additionally, the current study aimed to identify a group of serological biomarkers that may be used to monitor patients with BC during the course of the disease.

## Materials and methods

### Study cohort

The present study involved 96 recently diagnosed patients with BC who attended the National Cancer Institute (NCI) at Cairo University, (Cairo, Egypt) between September 2009 and October 2012. All patients were clear of distant metastasis at the initial diagnosis. Based on histological and immunophenotypical assessment of tumor samples, patients were separated into the following groups: i) TNBC, n=43, mean age=51.91±12.34 years, range=30–78 years; and ii) non-TNBC, n=53, mean age=52.77±12.13 years, range=27–81 years. Twenty healthy females who were age-matched (mean age=35±13.94 years; range, 22–64 years) were also included in the study as the controls.

Written informed consent was obtained from all participants prior to enrollment in the study. The Institutional Review Board of the NCI approved the study protocol, which was in accordance with the 2007 Declaration of Helsinki.

### Inclusion criteria

Patients enrolled in the study were ≥18 years old; presented with histologically-confirmed BC (TNBC or non-TNBC); had an Eastern Cooperative Oncology Adequate performance: ≤2 (19); and exhibited adequate bone marrow (WBC count, ≥3.0×10^9^/l; ANC, ≥1.5×10^9^/l; platelet count, ≥100×10^9^/l; hemoglobin level, ≥9 g/l), liver (serum bilirubin, <1.5×ULN; ALT and AST levels, <3 times normal values) and kidney (plasma creatinine level, <1.5 times normal value) function. Exclusion criteria included pregnancy, breast-feeding, an active secondary malignancy or involvement in another clinical trial.

### Treatment and follow-up

Patients received FEC100 as follows: Cyclophosphamide (Baxter, Deerfield, IL, USA) 500 mg/m^2^ IV diluted in 50 ml of normal saline as a 5- to 10-minute intravenous infusion on day 1; Epirubicin (Pfizer, New York, NY, USA) 100 mg/m2 IV diluted in 50 ml of normal saline as a 5- to 10-minute intravenous infusion on day 1; Fluorouracil (Ebewe Pharm, Unterach, Austria) 500 mg/m^2^ IV D1 as a bolus intravenous injection adjuvant for three cycles followed by 75 mg/m^2^ docetaxel (Sanofi-Aventis, Paris, France) for four cycles every 21 days with standard pre-medication (anti-emetics, anti-allergins and proton pump inhibitors). Radiotherapy was administered when indicated subsequent to the completion of chemotherapy (50 Gy in 2.0 Gy daily fractions) followed by hormonal therapy whenever indicated, according to the hormonal status of the tumor in ER and/or PR positive types of tumor. In the cases with metastasis, responses to treatment were assessed using the Response Evaluation Criteria in Solid Tumors system (20) and accordingly, patients were categorized into the following groups: i) Complete response (CR), complete disappearance of disease confirmed at 4 weeks; ii) partial response (PR), 30% reduction; iii) stable disease (SD), neither CR nor PR criteria were fulfilled; and iv) progressive disease (PD) presence of metastasis and/or recurrence observed as a 20% increase in tumor measurements or appearance of new lesions. The median follow-up period was 33 months. Local recurrence and distant metastases were assessed; and disease-free survival (DFS) and overall survival (OS) were calculated.

### Blood acquisition and serum preparation

A total of 5 ml venous blood was collected in a sterile 15 ml plastic Falcon tube (Becton-Dickinson, Franklin Lakes, NJ, USA), was left to clot and was then centrifuged at 11,000 × g for 15 min. Serum samples were stored at −80°C until they were required for the assay.

### Detection of VEGF-A, TGF-β1, IGF-I and IGF-IR expression in sera

Quantitative enzyme-linked immunosorbent assay (ELISA) kits were used to assess the levels of human IGF-I (cat. no. EIA-4140; DRG Instruments GmbH, Marburg, Germany), IGF-IR (cat. no. # OK-0226; Uscn Life Sciences Inc., Missouri, TX, USA), VEGF-A (cat. no. BMS277/2; eBioscience Bender Medsystems GmbH, Vienna, Austria) and TGF-β1 (cat. no. EIA-1864; DRG Instruments GmbH) according to the manufacturer’s instructions. A total of 100 μl (for VEGF-A, IGF-I and IGF-IR) or 200 μl (for TGF-β1) of prediluted sera was added to micro-titer wells precoated with anti-human IGF-I monoclonal, anti-human IGF-IR monoclonal, anti-human VEGF-A polyclonal and anti-human TGF-B1 polyclonal antibodies followed by a biotin-conjugated anti-human IGF-I, IGF-IR, VEGF-A, mouse anti-TGF-β1 antibodies and streptavidin-horseradish peroxidase. Color was developed using a tetramethyl benzidine-hydrogen dioxide mixture and terminated with sulfuric acid. The absorbance of each well was determined using a spectrophotometer (PR 3100 TSC Microplate Reader, Bio-Rad, Hercules, TX, USA).

### Statistical analysis

SPSS, version 20.0 (IBM SPSS, Armonk, NY, USA) was used for statistical analysis and data were expressed as the mean rank or mean ± standard deviation for continuous variables. Categorical variables were assessed using the χ^2^ test when appropriate. All P-values are two-tailed and P<0.05 was considered to indicate a statistically significant difference. Comparisons of the markers between different groups were performed using the Mann-Whitney U test or Kruskal Wallis one-way analysis of variance. The associations between TNBC, clinicopathologic variables and the significance of markers were examined using the χ^2^ test. The association with survival was analyzed using Kaplan-Meier analysis and curves were compared using the log-rank test and Cox regression analysis to adjust for other prognostic indicators. The Receiver Operating Characteristic (ROC) curve was used to determine the appropriate cut-off level of markers in the diagnosis of TNBC.

## Results

### Patient characteristics

In general, the patients with TNBC presented with more aggressive tumors compared with those with non-TNBC, with a higher incidence of lymph nodes metastasis (P<0.001), recurrence (P<0.001) and distant metastasis (P<0.001). Patients with TNBC exhibited significantly lower response rates compared with the patients with non-TNBC (P<0.001) (Table I).

### Marker expression in the sera of patients and controls

**Figure 1:**
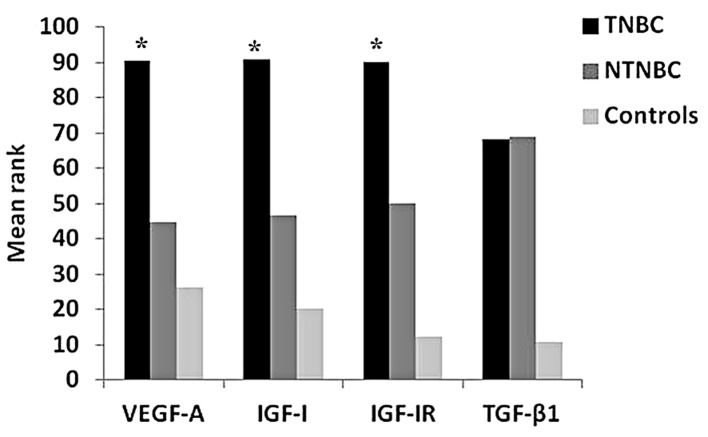
Mean rank of the markers in the TNBC, non-TNBC and control groups. TGF-β1, transforming growth factor-β1; IGF-I, insulin-like growth factor I; IGF-IR, IGF-I receptor; VEGF-A, vascular endothelial growth factor-A; TNBC, triple-negative breast cancer.

The serum level mean ranks for VEGF-A were 90.49, 44.73 and 26.22; for IGF-I were 90.72, 46.80 and 20.23; for IGF-IR were 90.20, 50.17 and 12.40; and for TGF-β1 were 68.01, 68.76 and 10.85, in the TNBC, non-TNBC and NC groups, respectively. The differences between the serum levels of VEGF-A, IGF-I and IGF-IR in the three groups were all statistically significant (P<0.001). The level of serum TGF-β1 differed significantly between the control group and the other two groups (P<0.001), but not between TNBC and non-TNBC (P=0.282) (Fig. 1 and Table II).

**Figure 2:**
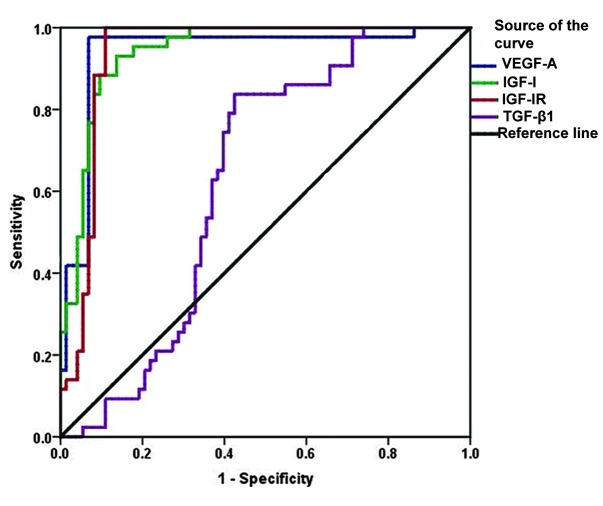
ROC curve analysis for markers in triple-negative compared with non-triple-negative and normal control to calculate the optimal cut-off value. ROC, receiver operating characteristic; VEGF-A, vascular endothelial growth factor-A; IGF-I, insulin-like growth factor I; IGF-IR, IGF-I receptor; TGF-β1, transforming growth factor-β1.

**Figure 3:**
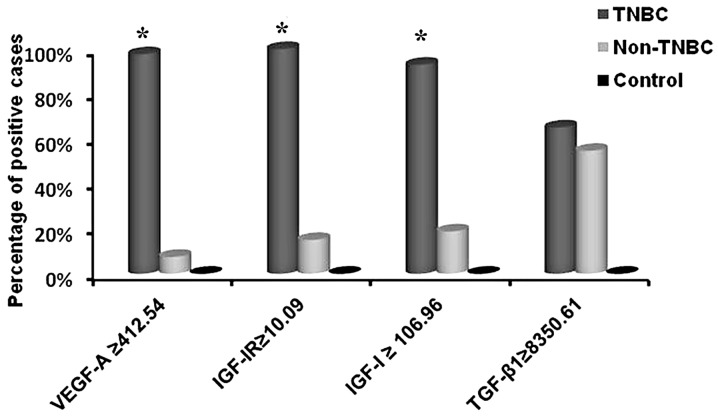
Percentage of positive cases of each studied marker in different investigated groups. TNBC, triple-negative breast cancer; VEGF, vascular endothelial growth factor; IGF-I, insulin-like growth factor I; IGF-IR, IGF-I receptor; TGF-β1, transforming growth factor-β1.

The cutoffs identified by ROC curve analysis that were able to differentiate between the patients with TNBC and non-TNBC were 106.96 ng/ml for serum IGF-I (93% sensitivity and 86.3% specificity), 10.09 ng/ml for serum IGF-IR (100% sensitivity and 89% specificity) and 412.54 pg/ml for serum VEGF-A (97.7% sensitivity and 94.2% specificity) (Fig. 2). The positivity rates of IGF-I (93%), IGF-IR (100%) and VEGF-A (92.7%) were significantly higher in the TNBC group compared with non-TNBC group(P<0.001; Fig. 3).

### Correlation between the markers

Significant correlations were identified between the serum levels of IGF-IR and IGF-I (r=0.645; P<0.001), VEGF-A (r=0.594; P<0.001) and TGF-β1 (r=0.307; P=0.001). Serum VEGF-A and IGF-I were also positively correlated (r=0.511; P<0.001) (Table III).

### Correlations between serological markers and clinicopathological features of the patients

A significant association was identified between high serum levels of VEGF-A and large tumor size (P=0.02) whereas high TGF-β1 was associated with positive HER2 expression (P=0.043). High levels of serum VEGF-A, IGF-I and IGF-IR were significantly associated with the presence of lymph nodes metastasis (P=0.007, P=0.007 and P=0.03, respectively) and incidence of mortality (P<0.001, P=0.009 and P=0.03, respectively). High serum levels of VEGF-A, IGF-I, IGF-IR and TGF-β1 were significantly associated with a high incidence of recurrence (P<0.001, P<0.001, P<0.001 and P=0.02, respectively), the presence of distant metastasis (P=0.026, P<0.001, P<0.001 and P=0.02, respectively) in TNBC and non-TNBC patients and lower rate of response (P=0.004, P<0.001, P=0.003 and P<0.001) in metastatic BC patients (Table IV). In the TNBC group, high serum levels of TGF-β1 were associated with development of distant metastasis (P=0.009), recurrence (P=0.003) and poor response to treatment (P= 0.01), whereas, high serum levels of IGF-I were associated with metastasis (P=0.026), recurrence (P=0.01), mortality rate (P=0.03) and impaired response (P= 0.037; Table V).

### Response to treatment

During the period of follow-up, 90 (41 TNBC and 49 non-TNBC) out of the 96 cases were assessed for disease progression. At the conclusion of the study (median follow up, 33 months; range, 2–85 months), 41/90 (45.6%) of the patients exhibited disease progression (presence of metastasis and/or recurrence). Local recurrence occurred in 28/41 (68.3%) of the TNBC group compared with 9/49 (18.40%) of the non-TNBC group (P<0.001) (Table I). BC recurrence was associated with large tumor size 21/37 (56.80%; P=0.046), lymph node metastasis 35/37 (94.60%; P<0.001) and distant metastasis 34/37 (91.90%; P<0.001). The overall response rate was 35.9% (14/39, 95% CI, 20.5–51.3%) and was significantly lower in patients with TNBC (Table I). Patients with PD exhibited higher serum levels of VEGF-A, IGF-I, IGF-IR and TGF-β1, compared with those who responded or had SD (P=0.004, P=0.001, P=0.003 and P<0.001, respectively; Table IV).

### Survival analysis

**Figure 4:**
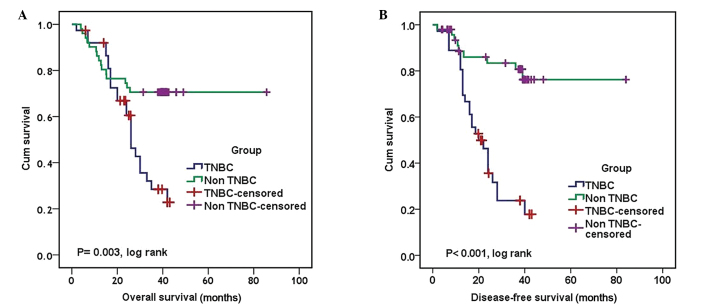
Kaplan-Meier analysis. (A) Overall survival and (B) disease-free survival of the triple- and non-triple-negative patients with breast cancer. TNBC, triple-negative breast cancer.

A total of 40/90 patients (44.4%) succumbed to the disease over the course of the study and of these, 63.40% were classified as TNBC and 28.60% were non-TNBC patients (Table II). TNBC patients showed poor prognosis in terms of OS (P= 0.003, log rank) and DFS (P<0.001, log rank) compared to non-NTNBC patients (Fig. 4). The median OS and DFS for patients with TNBC were 26 and 20 months, respectively (95% CI, 22.09–29.91 and 14.52–24.75 months, respectively). The median OS and DFS for patients with non-TNBC could not be defined as >50% of patients were alive and free of recurrence at the conclusion of the study (P=0.003 and P<0.001, log rank, respectively) (Table VI and Fig. 4).

**Figure 5:**
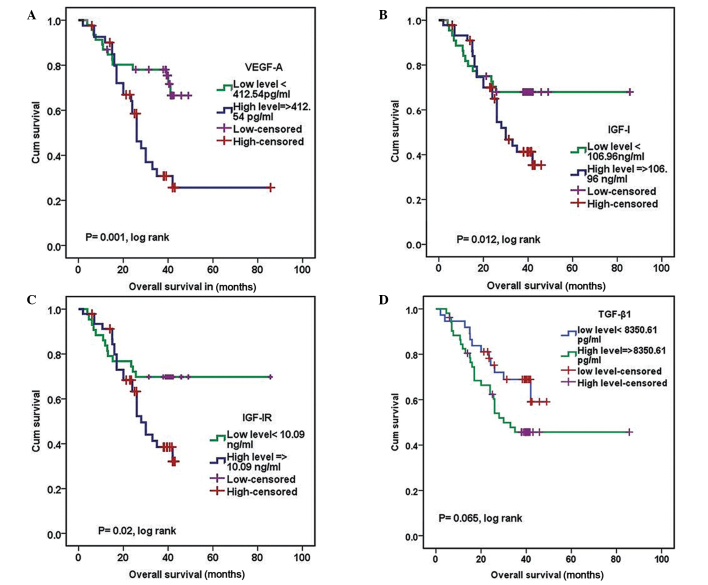
Overall survival analysis for (A) VEGF-A, (B) IGF-I, (C) IGF-IR and (D) TGF-β1 expression. VEGF-A, vascular endothelial growth factor-A; IGF-I, insulin-like growth factor I; IGF-IR, IGF-I receptor; TGF-β1, transforming growth factor-β1.

**Figure 6:**
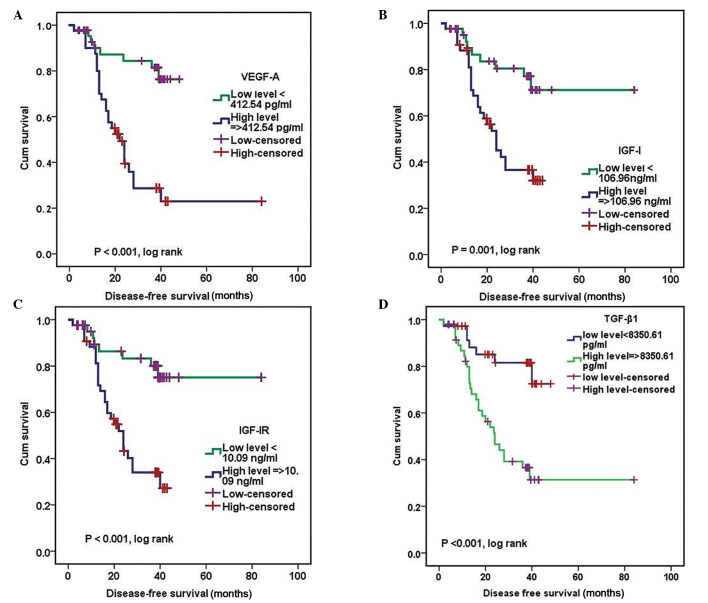
Disease-free survival analysis for (A) VEGF-A, (B) IGF-I, (C) IGF-IR and (D) TGF-β1 expression. VEGF-A, vascular endothelial growth factor-A; IGF-I, insulin-like growth factor I; IGF-IR, IGF-I receptor; TGF-β1, transforming growth factor-β1.

Patients with positive expression of VEGF-A, IGF-I and IGF-IR experienced poorer outcomes in terms of OS (P=0.001, P=0.012 and P=0.012, log rank, respectively) and those with positive expression of four studied markers were associated with reduced DFS (Fig. 5 and Fig. 6) A univariate analysis demonstrated that reduced OS was associated with TNBC (P=0.003), high serum levels of VEGF-A (P=0.003), IGF-I (P=0.03), IGF-IR (P=0.02), large tumor size (P=0.007), and lymph nodes metastasis (P=0.004) in the overall population (Table VI). Reduced DFS was significantly associated with high serum levels of VEGF-A, TGF-β1, IGF-IR (P<0.001) and IGF-I (P=0.003), high tumor grade (P=0.01; tumor grade indicates the aggression of the tumor), lymph nodes metastasis (P=0.004) and large tumor size (P=0.008; Table VI).

In the multivariate analysis, only positive lymph nodes (P=0.01; HR, 14.68; 95% CI, 1.8–104.6) and high serum levels of VEGF-A (P=0.005; HR, 1.001; 95% CI, 0.2–1.14), IGF-I (P=0.044; HR, 2.0; 95% CI, 1.019–3.4) and IGF-IR (P=0.048; HR, 1.98; 95% CI, 1.03–3.84) were independent factors for OS. Tumor size, lymph nodes invasion and TGF-B1 were independent predictors for DFS.

## Discussion

The prognostic outcome of TNBC should be discussed with regard to specific molecular subgroups. Previous studies have focused on the prognostic and predictive values of circulating tumor-associated markers due to the use of these biomarkers being cheap, non-invasive and non-specific to disease stage (21).

In the current study, compared with patients with non-TNBC, TNBC patients were observed to present with more aggressive tumors with lymph nodes invasion, distant metastases, low response to treatment and a high incidence of early recurrence and cancer-associated mortality, which was consistent with previous studies (22–24). The serum levels of VEGF-A, IGF-I and IGF-IR, but not TGF-β1 were identified at specific cutoffs as potential surrogate markers to differentiate between TNBC and non-TNBC, and to better sub-classify TNBC into prognostic subgroups, since their expression was significantly higher in the TNBC group compared with non-TNBC groups and healthy controls (P<0.001).

The current study demonstrated that high serum levels of TGF-β1 were significantly associated with a high incidence of metastasis, recurrence and a poor response to treatment, which was consistent with previous studies (21,25). It was demonstrated that although BC tumor tissue exhibits higher levels of expression of TGF-β1 than the corresponding normal tissues, the association of TGF-β1 with cancer is strongest in the advanced stages of the disease. One possible explanation is that TGF-β1 signaling has dual tumor suppressive and metastatic roles in BC. In the early stages, TGF-β1 suppresses tumor development by maintaining the balance between cell renewal and cell differentiation or loss (26). Loss of this homeostatic function occurs early in carcinogenesis (27) then, two important alterations occur during tumor development. The first alteration is associated with a global loss of receptor signaling, resulting in a reduction in the tumor suppressive activity of TGF-β1, whereas the second is associated with overproduction of bioactive TGF-β1, resulting in the activation of a pro-invasive, -angiogenic and -metastatic TGF-β1-regulated gene expression program, thus inducing a tumor cell phenotype that is mesenchymal and highly metastatic (11). This hypothesis provides an explanation for the association between TGF-β1 overexpression and the increased incidence of metastasis and recurrence reported in the current study. A reduction in the expression of the TGF-β1 receptor (TGF-β1-R) is associated with an increase in the levels of TGF-β1 in the tumor microenviroment and abrogates the tumor-suppressive effects of TGF-β1 and the invasive phenotype in the majority of cases of BC.

It was reported that ER negative BC cells expressed TGF-βR which were undetectable in ER positive cells. Moreover, It has been shown that, the differentiated ER positive luminal cells are unresponsive to TGF-β, since the TGF-βR-II gene is transcriptionally silent in these cells (28). They respond to estrogen via down regulation of TGF-β, whereas anti-estrogens act by upregulating TGF-β1 signal transduction pathway (29). Thus, the inhibition of TGF-β1 signaling results in the differ entiation of mammary stem cells into ductal cells. Accordingly, the TGF-β1 antagonists convert basal-like or HER2-positive tumor cells into epithelioid, non-proliferating and non-metastatic cells, which makes them candidates for targeted therapy in the TNBC cases (11).

TGF-β1 signaling also induces macro-metastases, particularly in the bones and lungs (20–32). In a mouse model of TNBC or basal-like BC, treatment with TGF-β1 neutralizing antibodies or receptor kinase inhibitors strongly inhibited the development of distant metastases. This was via the derepression of antitumor immunity, inhibition of angiogenesis or the reversal of the mesenchymal, invasive phenotype characteristic of HER2-positive and basal-like BC (11). Additionally, TGF-β1 may autonomously promote metastasis, as the expression of a dominant-negative mutant of TGF-βR-II in the TNBC cell line MDA-MB-231 was reported to inhibit experimental bone metastases, whereas the overexpression of constitutively active TGF-βR-I increased the production of parathyroid hormone-related protein by the tumor cells and enhanced bone metastases (33). In addition, VEGF-A, -B and -C were observed to be upregulated in bone and bone marrow metastases compared with those in the brain or lung (34).

The prognostic impact of TGF-β1 in BC remains controversial. Certain studies have demonstrated that TGF-β1 overexpression is significantly higher in patients with favorable outcomes compared with those with a poor prognosis (35), whereas others indicated the reverse (36,37). The results of the current study regarding the poor prognostic impact of TGF-β1 overexpression are consistent with those of Ivanović *et al* (21) and Dave *et al* (38), who observed increased levels of plasma TGF-β1 in locally advanced BC (stages III and IV). In addition to the observation by Dave *et al* (38) who reported a correlation between low serum TGF-β1 levels and pathological CR and prolonged DFS

In the present study, VEGF-A was observed to be significantly overexpressed in TNBC compared with non-TNBC. It was also associated with aggressive tumors, lymph nodes invasion, a high incidence of metastasis, poor response to treatment and reduced survival. These observations are comparable to those of previous studies on metastatic (39) and non-metastatic (40,41) TNBC in which VEGF-A was demonstrated to be important in the progression of TNBC. As a key mediator of angiogenesis, VEGF-A stimulates the proliferation and migration of epithelial cells, inhibits apoptosis of endothelial tissues and increases vascular permeability and vasodilation (42). In accordance with this, the current study reported low VEGF-A levels in tumors that were responsive (CR and PR) compared with those that were nonresponsive (SD and PD) (P=0.004) to chemotherapy, and this was also associated with prolonged survival. Similar results were reported previously by Björndahl *et al* (43), who suggested that IGF-IR is able to induce metastasis via the regulation of tumor cell survival and proliferation in secondary sites, in addition to the promotion of angiogenesis and lymphangiogenesis either through direct action on the endothelial cells or by transcriptional regulation of VEGF-A and -C.

IGF-IR, a member of a transmembrane receptor tyrosine kinase family, is expressed on the cell surface of cells in the majority of tissues. Together with its ligand (IGF-I), it is important in the regulation of cell cycle progression, cell survival and apoptosis (16,17,44–47). Although several multi-center studies have demonstrated that serum IGF-I predicts the outcome of patients with BC (48–50) and others (51,52) observed the correlation between high IGF-I mRNA levels and longer OS and DFS in cases of BC, this was not assessed in TNBC. Thus, to the best of our knowledge, this is the first study to investigate these factors in TNBC.

High levels of IGF-IR were detected in 100% of the TNBC cases. Previous studies reported IGF-IR expression in 29–36% of TNBC (53) and in certain studies IGF-IR overexpression in TNBC was attributed to either mutations in tumor suppressor genes, including p53 and BRCA1, which repress the IGF-IR promoter (54), or to the amplification of IGF-IR in basal or HER-2 positive BC. However, these were not assessed in the current study. A significant correlation between IGF-I/IGFR-IR and VEGF-A expression was demonstrated in the current study, and the contribution of these markers to an aggressive BC phenotype was confirmed. Serum IGF-IR levels were demonstrated to be significantly lower in patients who experienced complete and partial responses compared with those with PD and SD (P=0.003). In addition, high serum IGF-I/IGF-IR levels were significantly associated with reduced OS, independent of other clinicopathological features. Concerning this observation, Haffner *et al* (51) demonstrated that the IGF-I mRNA level was an independent predictor of OS and DFS in 89 lymph-node-negative cases of BC. Additionally, Shin *et al* (52) measured IGF-I and IGF-IR mRNA levels in 508 breast tumors and adjacent tissues, and observed that patients in the highest tertile of tumor IGF-I mRNA levels exhibited a longer DFS and OS compared with those in the lower tertile.

One hypothesis is that although a number of studies regarding cancer cell lines have indicated that IGF-I stimulation leads to aggressive, fast growing, metastasizing tumors, other studies demonstrate that IGF-I is also able to increase cell differentiation in certain cancer cell lines that are associated with less aggressive types of cancer and hence improved prognosis. One explanation is that IGF-I expression may be a by-product of another cellular process that results in a less aggressive phenotype. An additional possibility is that unlike serum IGF-I levels, the expression of IGF-I in breast tissue may not be in large enough quantities to result in an increase in stimulation of the IGF-IR signaling pathway (16,55). However, until verified by larger studies, IGF-I expression in tissue and the serum should be used as an intermediate prognostic marker, rather than a potential therapeutic target.

By contrast, Munagala *et al* (56) demonstrated that IGF-I and TGF-β1 act synergistically to induce epithelial-mesenchymal transition in BC cells, leading to metastasis. It was identified that IGF-I transmits signals via the phosphoinositide 3-kinase and mitogen-activated protein kinase pathways resulting in the transcription of unknown but specific genes. The products of these genes result in the extracellular activation of IGF-IR, which stimulate cancer cell proliferation and survival, and confer resistance to cytotoxic, hormonal and targeted therapies in BC. In accordance with this, Munagala *et al* concluded that IGF-I and TGF-β1 are promising molecular targeted therapies in BC.

Shimizu *et al* (57) also observed IGF-IR overexpression in BC biopsies while Pizon *et al* (58) and Munagala *et al* (56) demonstrated that IGF-IR may serve an important role in determining how aggressive circulating tumor cells (CTCs) are and their ability to grow subsequent to adhesion to form metastatic deposits. Patients with high CTC counts were demonstrated to commonly exhibit high IGF-IR expression on the tumor cells and in the CTCs. In addition, a significant linear correlation was reported between IGF-IR expression and the presence of VEGFR-2 on the isolated CTCs.

Through the multivariate analysis, serum IGF-I, IGF-IR and VEGF-A levels were identified as independent predictors for OS together with lymph nodes invasion and TNBC, whereas only TGF-β1, large tumor size and lymph nodes metastasis were independent predictors for DFS. Similar results were obtained by Grau *et al* (59), who observed that patients with the highest circulating levels of TGF-β1 often have reduced survival independent of the disease stage, whereas additional studies demonstrated that TGF-β1 levels were significantly higher in patients with a favorable outcome (36,37). These discrepancies may be due to the small sample sizes and the relatively short follow-up periods in the majority of studies.

In conclusion, to the best of our knowledge, the current study was the first to simultaneously assess serum IGF-I, IGF-IR, VEGF-A and TGF-β1 levels and their interrelations in two well defined groups of patients with BC (TNBC and non-TNBC). The results of the present study confirm that high expression of these proteins is more common in patients with TNBC and is usually associated with an aggressive tumor phenotype with higher incidence of recurrence, poor response to treatment and reduced survival. Therefore, TGF-β1, IGF-I/IGF-IR and VEGF-A may be promising surrogate prognostic markers and possibly candidates for targeted therapy, particularly in patients with TNBC. However, large clinical trials are required in order to verify the results of the current study.

## Figures and Tables

**Table I:** Clinicopathological features of the patients with triple-negative and non-triple-negative breast cancer (descriptive statistics).

Patient characteristic	Total no.	Triple-negative, % (n) total=43	Non-triple-negative, % (n) total=53	Statistics
Age at diagnosis (years)				
≤35	8	9.3 (4)	7.55 (4)	χ^2^=0.32
36–49	34	32.56 (14)	37.74 (20)	P=0.85
≥50	54	58.14 (25)	54.71 (29)	
Tumor size (cm)				
<4	52	44.2 (19)	62.30 (33)	χ^2^=3.13
≥4	44	55.8 (24)	37.70 (20)	P=0.071
Family history				
No	85	95.30 (41)	83.02 (44)	χ^2^=3.40
Yes	11	4.70 (2)	16.98 (9)	P=0.065
Menopausal status				
Pre-menopausal	44	44.19 (19)	47.17 (25)	χ^2^=0.085
Post-menopausal	52	55.81 (24)	52.83 (28)	P=0.71
Tumor stage				
Early (I-II)	55	60.50 (26)	54.70 (29)	χ^2^=0.50
Late (III-IV)	41	39.50 (17)	45.30 (24)	P=0.48
Tumor grade				
1	2	0.00 (0)	3.80 (2)	χ^2^=3.13
2	79	79.07 (34)	84.90 (45)	P=0.28
3	15	20.90 (9)	11.30 (6)	
Nodal status				
Negative	26	6.98 (3)	43.40 (23)	χ^2^=15.90
Positive	70	93.02 (40)	56.70 (30)	P<0.001
Metastasis				
No	51	30.77 (12/39)	76.47 (39/51)	χ^2^=18.80
Yes	39	69.23 (27/39)	23.53 (12/51)	P<0.001
Estrogen receptor status				
Positive	38	0.00 (0)	71.70 (38)	χ^2^=51.03
Negative	58	100 (43)	28.30 (15)	P<0.001
Progesterone receptor status				
Positive	31	0.00 (0)	58.50 (31)	χ^2^=37.15
Negative	65	100 (43)	41.50 (22)	P<0.001
HER2 status				
Positive	18	0.00 (0)	33.96 (18)	χ^2^=17.97
Negative	78	100 (43)	66.04 (35)	P<0.001
Breast cancer recurrence				
No	53	31.70 (13/41)	81.60 (40/49))	χ^2^=22.98
Yes	37	68.30 (28/41)	18.40 (9/49)	P<0.001
Mortality				
No	50	36.60 (15/41)	71.40 (35/49)	χ^2^=9.20
Yes	40	63.40 (26/41)	28.60 (14/49)	P=0.002
Response to treatment				
Responsive (CR+PR)	14	14.81 (4/27)	83.33 (10/12)	χ^2^=19.80
Nonresponsive (SD+PD)	25	85.19 (23/27)	16.67 (2/12)	P<0.001

Patient’s followed up for survival and recurrence were 90 (41 TNBC and 49 NTNBC). Patient’s followed up for response were 39 (27 TNBC and 12 NTNBC).

**Table II:** Serum levels of VEGF-A, IGF-I, IGF-IR and TGF-β1.

Group	VEGF-A		IGF-I		IGF-IR		TGF-β1	
	Mean rank	P-value	Mean rank	P-value	Mean rank	P-value	Mean rank	P-value
TNBC	90.5^a^^b^		90.7^a^^b^		90.2^a^^b^		68.0^a^	
Non-TNBC	44.7^a^	<0.001	46.8^a^	<0.001	50.2^a^	<0.001	68.8^a^	<0.001
Control	26.2		20.2		12.4		10.9	

a vs. control;

b vs. non-TNBC. TNBC, triple-negative breast cancer; VEGF-A, vascular endothelial growth factor-A; IGF-I, insulin-like growth factor I; IGF-IR, IGF-I receptor; TGF-β1, transforming growth factor-β1.

**Table III:** Pearson bivariate analysis r-values of serum levels of VEGF-A, IGF-I, IGF-IR and TGF-β1.

	VEGF-A	IGF-I	IGF-IR	TGF-β1
VEGF-A	1	0.511	0.594	0.116
P-value		<0.001	<0.001	0.22
IGF-I	0.511	1	0.645	0.121
P-value	<0.001		<0.001	0.20
IGF-IR	0.594	0.645	1	0.307
P-value	<0.001	<0.001		0.001
TGF-β1	0.116	0.121	0.307^b^	1
P-value	0.22	0.22	0.001	

VEGF-A, vascular endothelial growth factor-A; IGF-I, insulin-like growth factor I; IGF-IR, IGF-I receptor; TGF-β1, transforming growth factor-β1.

**Table IV:** Correlation of the mean rank of markers to different clinicopathological parameters.

Characteristic	Total no.	VEGF-A mean rank	Statistics	IGF-I mean rank	Statistics	IGF-IR mean rank	Statistics	TGF-β1 mean rank	Statistics
Age at diagnosis (years)									
≤35	8	42.81	χ^2^=0.66	49.81	χ^2^=0.19	45.69	χ^2^=0.12	51.00	χ^2^=0.23
36–49	34	51.06	P=0.72	46.84	P=0.91	48.10	P=0.94	49.76	P=0.89
≥50	54	47.73		49.35		49.17		47.33	
Tumor size (cm)									
<4	52	40.56	χ^2^=5.47	42.8	χ^2^=2.1	42.52	χ^2^=2.60	45.57	χ^2^=0.08
≥4	44	53.57	P=0.02	50.90	P=0.15	51.24	P=0.12	47.61	P=0.72
Family history									
No	85	48.53	χ^2^=7.70	47.63	χ^2^=3.2	47.23	χ^2^=3.40	45.78	χ^2^=0.05
Yes	11	22.94	P=0.006	31.11	P=0.07	34.78	P=0.18	48.00	P=0.81
Menopausal status									
Pre-menopausal	44	47.72	χ^2^=0.07	47.57	χ^2^=0.09	48.90	χ^2^=0.017	48.22	χ^2^=0.005
Post-menopausal	52	49.16	P=0.8	49.29	P=0.76	48.16	P=0.9	48.74	P=0.93
Tumor stage									
Early (I–II)	55	47.99	χ^2^=1.60	45.77	χ^2^=0.093	43.56	χ^2^=0.38	46.31	χ^2^=0.32
Late (III–IV)	41	40.80	P=0.2	43.92	P=0.74	47.03	P=0.53	43.16	P=0.57
Tumor grade									
1	2	39.25	χ^2^=0.24	21.50	χ^2^=3.96	46.50	χ^2^=2.59	77.5	χ^2^=1.58
2	79	46.60	P=0.61	46.28	P=0.13	45.58	P=0.27	47.80	P=0.21
3	15	53.71		58.04		58.32		41.54	
Nodal status									
Negative	26	31.54	χ^2^=8.20	31.50	χ^2^=8.21	33.72	χ^2^=4.8	48.96	χ^2^=1.57
Positive	70	47.87	P=0.007	47.88	P=0.007	47.07	P=0.03	41.51	P=0.22
Metastasis									
No	51	40.14	χ^2^=4.60	36.75	χ^2^=10.80	36.96	χ^2^=11.60	40.02	χ^2^=5.20
Yes	39	52.51	P=0.026	56.94	P<0.001	56.67	P<0.001	52.67	P=0.02
Estrogen receptor status									
Positive	38	26.89	χ^2^=27.40	26.26	χ^2^=28.20	27.53	χ^2^=24.80	47.44	χ^2^=0.32
Negative	58	57.35	P<0.001	57.75	P<0.001	56.94	P<0.001	44.26	P=0.57
Progesterone receptor status									
Positive	31	24.07	χ^2^=28.99	27.98	χ^2^=20.31	28.68	χ^2^=19.10	46.71	χ^2^=0.06
Negative	65	55.18	P<0.001	53.41	P<0.001	53.10	P<0.001	44.95	P=0.77
HER2 status									
Positive	18	32.60	χ^2^=4.76	26.40	χ^2^=9.30	27.67	χ^2^=18.70	57.00	χ^2^=4.09
Negative	78	48.08	P=0.03	49.32	P=0.002	49.07	P<0.001	43.20	P=0.043
Breast cancer recurrence									
No	53	35.11	χ^2^=13.50	34.41	χ^2^=15.80	35.47	χ^2^=12.40	40.00	χ^2^=5.20
Yes	37	55.15	P=<0.001	56.13	P=<0.001	54.65	P=<0.001	53.00	P=0.02
Mortality									
No	50	37.98	χ^2^=12.20	40.14	χ^2^=6.77	41.20	χ^2^=4.70	46.49	χ^2^=0.001
Yes	40	57.58	P=<0.001	54.76	P=0.009	53.38	P=0.03	46.51	P=0.99
Response to treatment									
Responsive (CR+PR)	14	13.10	χ^2^=8.30	11.90	χ^2^=11.1	12.80	χ^2^=8.70	11.07	χ^2^=13.4
Nonresponsive (SD+PD)	25	23.80	P=0.004	24.70	P=0.001	24.01	P=0.003	25.00	P<0.001
Disease progression									
Progressive (PD)	24	23.60	χ^2^=8.30	24.60	χ^2^=11.1	24.00	χ^2^=8.70	24.90	χ^2^=13.4
Non-progressive (CR+PR+SD)	15	13.30	P=0.004	11.95	P=0.001	12.90	P=0.003	11.09	P<0.001

P-values were calculated using the Kruskal-Wallis one-way analysis of variance. VEGF-A, vascular endothelial growth factor A; IGF-I, insulin-like growth factor I; IGF-IR, IGF-I receptor; TGF-β1, transforming growth factor β1; HER2, human epidermal growth factor 2; CR, complete response; PR, partial response; SD, stable disease; PD, progressive disease. Patients followed up for recurrence and survival were 90, patients followed up for responses were 39.

**Table V:** Correlation of serum levels of markers in triple-negative breast cancer patients (n=43).

Characteristic	Total no.	VEGF	Statistics	IGF-I	Statistics	IGF-IR	Statistics	TGF-β1	Statistics
Age at diagnosis (years)									
≤35	4	769.6±161.5	F=2.05	429.8±223.6	F=2.8	11.78±1.4	F=0.24	11499.5±5341.8	F=0.41
36–49	14	1172.2±534.3	P=0.14	268.8±75.8	P=0.07	12.3±2.5	P=0.79	9910±3023.3	P=0.67
≥50	25	932.7±377.4		275.4±131		11.88±1.4		9947.4±3086.4	
Tumor size (cm)									
<4	19	814.5±317	F=6.7	284.2±153	F=0.02	11.6±1.1	F=1.7	9779.6±3012.4	F=029
≥4	24	1138.8±467.4	P=0.013	290.3±115.1	P=0.88	12.3±2.2	P=0.2	10317.1±3461.2	P=0.6
Menopausal status									
Pre-menopausal	19	10528±499.6	F=0.7	303.4±130.3	F=0.48	12.12±2.2	F=0.14	10295.4±3437.	F=0.15
Post-menopausal	24	945.5±379.9	P=0.4	275.1±133.8	P=0.49	11.9±1.4	P=0.71	9908.8±3146.6	P=0.7
Tumor stage									
Early (I-II)	26	946.2±485	F=0.8	283.2±136.4	F=0.07	11.8±1.4	F=0.81	9986.1±3108.5	F=0.053
Late (III)	17	1070.9±345.3	P=0.36	294.4±127.4	P=0.79	12.3±2.3	P=0.37	10222.6±3533.7	P=0.82
Tumor grade									
1	0		F=3.7		F=0.02		F=0.007		F=0.24
2	34	931.8±387.6	P=0.06	286.1±123.3	P=0.89	11.98±1.9	P=0.9	9953±3018	P=0.63
3	9	1236.1±539.8		293.4±167.5		12.04±1.3		10558±4164.4	
Nodal status									
Negative	3	760.7±335.8	F=0.94	315.4±101.5	F=0.14	12.8±2.1	F=0.01	9789.4±2965.6	F=0.22
Positive	40	1013.1±439.8	P=0.34	285.5±134	P=0.71	11.8±1.7	P=0.8	10227.2±3487	P=0.65
Metastasis									
No	27	1045.2±416	F=0.89	322.9±140.1	F=5.2	11.94±1.9	F=0.18	10892.8±3496.2	F=7.5
Yes	12	900.2±500.1	P=0.35	219.7±104.2	P=0.026	11.7±1.2	P=0.68	7951.4±1897.3	P=0.009
Breast cancer recurrence									
No	13	880.7±501.4	F=0.7	196.5±113.1	F=7.48	12.4±2.8	F=1.65	7478±1698.6	F=10.5
Yes	28	1013.3±400.3	P=0.41	321.4±121.3	P=0.01	11.6±1.03	P=0.21	11092.2±3319.4	P=0.003
Mortality									
No	15	879.1±434.8	F=1.36	235.6±129.8	F=5.1	12.6±2.5	F=3.3	9063.1±2857.6	F=3.42
Yes	26	1035.4±408.2	P=0.25	328.5±123.1	P=0.03	11.5±1.0	P=0.08	10926.4±3316.3	P=0.07
Response to treatment									
Responsive (CR+PR)	4	923.7±477.3	F=0.31	213.8±111.7	F=4.69	11.6±1.0	F=2.7	7887.7±1687.6	F=7.34
Nonresponsive (SD+PD)	23	1009.2±401.4	P=0.58	308.9±122.8	P=0.037	12.6±2.8	P=0.11	10861.2±3308.7	P=0.01

VEGF, vascular endothelial growth factor; IGF-I, insulin-like growth factor I; IGF-IR, IGF-I receptor; TGF-β1, transforming growth factor β1; CR, complete response; PR, partial response; SD, stable disease; PD, progressive disease. Data presented as the mean ± standard deviation. TNBC patients followed up for recurrence and survival were 41. TNBC patients followed up for response to treatment were 27.

**Table VI:** Univariate and multivariate cox regression for disease-free survival and overall survival analysis.

Factor	Overall survival			Disease-free survival		
	HR	95% CI	P-value	HR	95% CI	P-value
Univariate factor						
Age group	0.69	0.5–1.09	0.12	0.65	0.4–1.04	0.08
Tumor size	2.44	1.27–4.68	0.007	2.5	1.26–4.88	0.008
Menopausal status	0.69	0.37–1.3	0.25	0.56	0.28–1.1	0.09
Tumor grade	1.83	0.83–4	0.13	2.8	1.28–6.11	0.01
Tumor stage	1.41	0.74–2.67	0.296	1.83	0.94–3.6	0.08
LN status	18.2	2.55–133	0.004	19.4	2.6–142.6	0.004
Tumor group (TN vs NTN)	2.6	1.37–5	0.003	5.2	2.4–11.3	<0.001
TGF-β1	1.87	0.95–3.7	0.07	4.1	1.79–9.5	<0.001
VEGF-A	2.73	1.39–5.36	0.003	4.8	2.16–10.75	<0.001
IGF-I	1.86	0.97–3.59	0.03	3.2	1.49–6.85	0.003
IGF-IR	2.15	1.1–4.2	0.02	3.85	1.85–8.02	<0.001
Multivariate factor						
Tumor size	1.95	0.9–3.79	0.06	2.4	1.13–4.9	0.02
LN status	14.68	1.8–104.6	0.01	14.7	1.8–118	0.01
Group	1.8	0.84–3.74	0.13	1.5	0.09–24.4	0.77
TGF-β1				4.3	1.7–10.96	0.002
VEGF-A	1.001	0.2–1.14	0.005	2.6	0.4–16.9	0.3
IGF-I	2.0	1.019–3.4	0.044	0.52	0.06–4.4	0.54
IGF-IR	1.98	1.03–3.84	0.048	1.3	0.3–5.5	0.73

HR, hazard ratio; CI, confidence interval; LN, lymph node; TN, triple-negative; NTN, non-triple-negative; TGF-β1, transforming growth factor-β1; VEGF-A, vascular endothelial growth factor -A; IGF-I, insulin-like growth factor I; IGF -IR, IGF -I receptor.
